# Fatal Immunohaemolysis after the Consumption of the Poison Pax Mushroom: A Focus on the Diagnosis of the Paxillus Syndrome with the Aid of Two Case Reports

**DOI:** 10.3390/diagnostics9040130

**Published:** 2019-09-26

**Authors:** Andreas Stöver, Bettina Haberl, Claudia Helmreich, Werner Müller, Frank Musshoff, Helena Fels, Matthias Graw, Olwen Groth

**Affiliations:** 1 Institute of Forensic Medicine, University of Munich, Nussbaumstrasse 26, 80336 Munich, Germany; 2 Department of Clinical Toxicology, Klinikum Rechts der Isar, Technical University of Munich, 81675 Munich, Germany; 3 Institute of Clinical Chemistry/Blood bank, Klinikum Rechts der Isar, Technical University of Munich, 81675 Munich, Germany; 4 Forensic Toxicological Center (FTC) Munich, 80335 Munich, Germany

**Keywords:** mushroom poisoning, disseminated intravascular coagulation (DIC), autoimmune haemolytic anaemia (AIHA), auto-anti-e antibody, chronic hepatitis C, post-mortem toxicology, *Paxillus involutus* (poison pax; brown roll-rim)

## Abstract

This retrospective report focuses on the diagnosis of the Paxillus syndrome, based on two fatal cases of haemolysis following the consumption of *Paxillus involutus*. These mushrooms are still consumed regularly, despite earlier reports of life-threatening autoimmune haemolytic anaemia. Such cases are nevertheless rare, and thus far no toxin could be identified that causes this unusual form of mushroom poisoning. All these factors contribute to the difficulty in diagnosing the Paxillus syndrome. The following aspects support the diagnosis in the two cases presented here: Both patients consumed the mushroom oftentimes before, yet allegedly without ill effects. Symptoms occurred 2–3 h after the last consumption, exacerbating into circulatory collapse, multiorgan failure, and death. Disseminated intravascular coagulation was identified as cause of death by autopsy of patient 1. Patient 2 died of multiorgan failure, mainly hepatic. Our mycological analyses could identify the consumed mushroom in both cases as *Paxillus involutus*. Furthermore, we could exclude anticoagulants and several other drugs as trigger for the haemolysis by post-mortem toxicological analysis. However, findings in each of the two cases may have led to the haemolysis, independent of the consumption of *Paxillus involutus*. Patient 1 carried the anti-erythrocytic antibody, auto-anti-e. Patient 2 contracted chronic hepatitis C years prior to the current incident. Considering the rarity of the Paxillus syndrome, our findings suggest that these patients were particularly susceptible for haemolysis after consuming this mushroom over a prolonged period. Occurrence of the Paxillus syndrome may thus be restricted to regular consumers of *Paxillus involutus* mushrooms with an existing predisposition for haemolysis.

## 1. Introduction

The collection and consumption of wild fungi is common practice in many cultures. Proper knowledge of mushrooms is however of absolute necessity, to avoid the accidental ingestion of toxic species. The global fatality rate due to the consumption of poisonous mushrooms is at least one hundred persons per year, whereas this is likely to be an underestimated number [1]. Intoxications with amatoxin containing mushrooms are by far the most common cause for fatal mushroom poisonings [2,3,4], of which *Amanita phalloides* plays a leading role [5]. Amatoxins in *Amanita phalloides*, commonly referred to as the death cap mushroom, can cause potentially fatal hepatotoxicity in the consumer. The first clinical signs typically present 8-12 hours after ingestion, leading to irreversible organ damage and finally death. 

In contrast to amatoxin poisonings, reports of *Paxillus involutus* intoxications are very rare. Nevertheless, the effects of the latter may be just as life-threatening, making a correct diagnosis detrimental for positive patient outcome. Furthermore, because of similar clinical presentations, intoxications with the *Paxillus involutus* mushroom is often mistaken for amatoxin poisoning.

**Figure 1:**
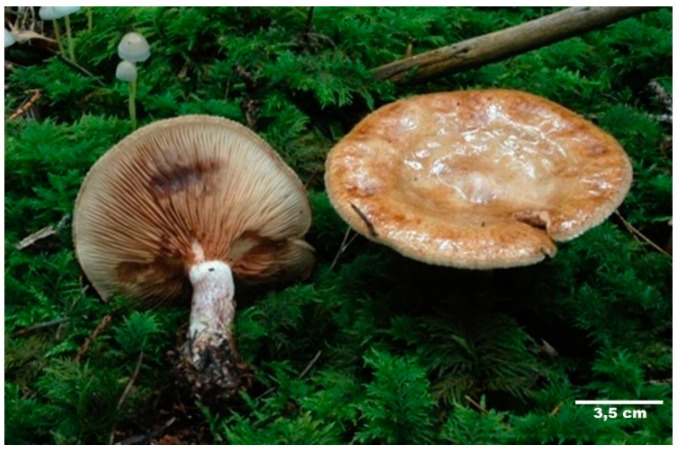
Image of *Paxillus involutus* (poison pax; brown roll-rim), photographed by mycologist and co-author, B. Haberl.

*Paxillus involutus*, commonly known as poison pax or brown roll-rim, has been widely consumed in earlier years. Meanwhile, following a number of fatal intoxications, the fungus is officially classified as dangerously poisonous [6,7,8,9]. Nevertheless, it is still considered edible by some mycophagists. The mushroom is widely distributed throughout the Northern Hemisphere and is known to spread in woody and grassy areas around late summer and autumn. It has, however, also been introduced to other parts of the world, including South Africa, Australia and South America. Its reddish, yellowish or olive-brown coloured funnel-shaped cap can grow up to 12 cm in diameter. The cap possesses a characteristic depressed centre with a distinctive in-rolled rim, with felt-like texture. Furthermore, the lamellae (gills) are decurrent and typically light in colour, but stain brown when touched or bruised (see Figure 1) [10]. 

Intoxications with brown roll-rim may present as one of two poisoning events, the first of which characteristically involves mainly gastrointestinal effects. This typically occurs after consuming the raw or insufficiently cooked mushroom, although similar reactions have occurred after proper heating [6]. Finally, usually all persons who consumed the mushrooms present with symptoms of this kind. The second poisoning event is known as the Paxillus syndrome. It affects only one of the consumers with autoimmune haemolytic anaemia (AIHA). Additional to gastrointestinal manifestations, symptoms may include subicterus, haemoglobinuria and pain around the lumbar spine. Disseminated intravascular coagulation (DIC) and multiorgan failure may cause death. Reports of the Paxillus syndrome are however rare. Our literature search for publications in the English and German languages resulted in merely fourteen cases of AIHA and hepatotoxicity associated with poison pax [4,6,7,8,9,11,12,13]. Six of these cases ended fatally [4,6,13].

Considering the rarity of the Paxillus syndrome and its unusual presentation as mushroom poisoning, we hereby provide a retrospective report of two fatal cases of AIHA, both of which occurred shortly after ingestion of *Paxillus involutus*. We discuss the challenges in identifying the condition and provide guidelines for successful diagnosis. The authors were not directly involved in the treatment of either of the two patients and wish to discuss these factors in retrospect from a forensic medical point of view. 

## 2. Methods

### 2.1. Case Histories and Course of Events

A short summary of the timeline of sequence of events between the consumption of the mushrooms and death of the two respective patients is given in Table 1.

#### 2.1.1. Case 1

A 46-year old Kazakhstani male died four days after consuming self-collected *Paxillus involutus* mushrooms. According to the available data, no coagulation disorders or other relevant diseases are included in his medical history. 

He and his wife oftentimes collected and consumed exclusively these mushrooms over the preceding ten years. Hitherto, allegedly neither showed any symptoms of poisoning nor intolerance. The mushrooms were cooked, marinated in vinegar, and refrigerated before consumption. Approximately three hours after consumption, the patient experienced severe pain around the lumbar spine and was thus transported to the local hospital. His wife remained asymptomatic. Increasing upper abdominal pain occurred, in the absence of vomiting and diarrhoea. Signs of inflammation increased with acute liver failure. 

Amatoxin poisoning due to the consumption of death cap mushrooms was considered. The patient was thus transferred to a maximum-care hospital, where treatment with silibinin and tazobactam was initiated. The consumed mushroom was meanwhile identified as *Paxillus involutus* by a mycologist. Amatoxin poisoning could thus be excluded and the silibinin therapy terminated. 

DIC occurred, affecting several organs. Computer tomography (CT)-scans of the thorax, abdomen and cranium were normal. No blood transfusion or haemofiltration was performed. Catecholamine-dependent circulatory insufficiency and severe septic shock occurred three days after the patient consumed the mushroom. Thrombocytopenia increased. Petechial haemorrhages appeared on the patient’s skin. Sudden hypertension occurred. CT-scan was now remarkable for massive cerebral swelling, leading to sinus thrombosis. 

The patient was declared brain dead and expired four days after mushroom ingestion. Medical records indicate the cause of death as sepsis and multiorgan failure of unknown origin.

#### 2.1.2. Case 2

A 32-year old Kazakhstani female, with a 19-year long history of chronic alcohol, nicotine and drug abuse, died three days after consuming *Paxillus involutus*. This patient had had no relation to patient 1. A history of heroin abuse was made known by a third party. No further details of recreational drug use are known. A hepatitis C infection was diagnosed 13 years prior, for which an interferon treatment was recorded only for the time of diagnosis. No current treatment was in effect, despite the prevailing infection. 

The deceased and family members regularly collected and ingested various types of wild mushrooms. Allegedly, none ever experienced intolerance. On this occasion, a combination of *Paxillus involutus*, honey fungus and porcini mushrooms were collected, cooked and fried, of which the patient and her parents ate. Two hours later, the patient became light-headed and sweated profusely, whereas her parents remained asymptomatic. Severe abdominal pain, nausea, vomiting and diarrhoea occurred. 

The patient was admitted to the local hospital one hour later. Activated charcoal was administered due to suspected mushroom intoxication. Circulatory insufficiency required admission of noradrenaline. Circulation and heart rate stabilised. Lactic acidosis was counteracted with sodium bicarbonate. A mycologist identified *Paxillus involutus* among the cooked mushrooms and hydrocortisone was administered to prevent anaphylactic shock.

**Figure 2:**
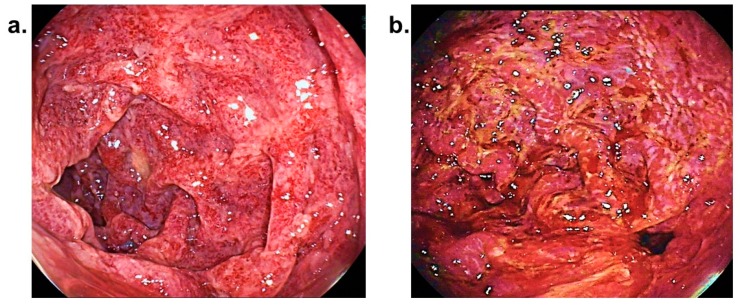
Images obtained during an oesophago-duodeno-gastroscopy of patient 2, showing (**b**) pronounced ischaemic changes of the mucous membranes of the gastric antrum and pylorus and (**a**) partial ischaemic, partial hyperaemic and partial infectious changes of the duodenal mucosa.

Persisting gross haematuria occurred. A bloody secretion appeared and persisted in the nasogastric tube. An oesophago-gastro-duodenoscopy was performed, during which the oesophagus appeared normal. However, significant damage of the mucous membranes in all areas of the stomach and visible areas of the duodenum could be located, as can be seen in a case of ischaemia (see Figure 2).

The patient was transported to a maximum-care hospital due to severe liver failure. Persistent coagulopathy, severe haemolysis and haemorrhagic gastroenteritis were treated with plasmapheresis. 

Atrial flutter and tachycardia occurred on day 2. Signs of cerebral oedema appeared. Plasmapheresis, renal replacement therapy and the application of haemo-adsorption filters led to stabilisation. However, the condition worsened during the next 24 h. Acute on chronic liver failure occurred. Liver transplantation was not an option due to the history of chronic alcoholism. Severe lactic acidosis persisted. Respiratory complications followed. 

The patient expired three days after hospital admission due to multiorgan failure, with leading hepatic failure.

### 2.2. Laboratory Tests during Hospitalisation and Post-Mortem Investigations

The presented laboratory values were obtained from patient hospital files. 

Standard procedures were followed for the autopsy of case 1. Post-mortem immunohaematological tests, mycological analyses and toxicological investigations were performed on femoral venous blood, gastric contents and urine samples, obtained during autopsy of case 1 and serum, obtained on day 2 of hospitalisation for patient 2. Due to the unavailability of patient samples, no histological investigations could be performed.

## 3. Results

### 3.1. Laboratory Tests During Hospitalisation

#### 3.1.1. Case 1

Meningitis, malaria and viral hepatitis could be excluded. Urine toxicological screening tested negative for amatoxins. Blood liver enzymes and inflammation parameters were highly elevated. A massive increase in lactate dehydrogenase (LDH) was indicative of severe haemolysis. No exact laboratory values are, however, available for these parameters. 

Haematological tests showed that the patient had blood group B, with Rhesus formula CcD.ee. Furthermore, the auto-anti-e antibody was identified. A direct Coombs test was strong positive (4+).

#### 3.1.2. Case 2

Patient blood was free of alcohol at the time of hospital admission. On day 2, the prothrombin time was indicative of severe coagulation deficits and the international normalized ratio (INR) and Quick value were 4.51 and 16%, respectively. These improved to 1.44 and 57% by the end of day 2. Haemoglobin decreased to 8.9 g/dL in ethylenediaminetetraacetic acid (EDTA) blood. Haptoglobin in patient serum declined from 760 mg/L on day 2 to below 150 mg/L on day 3. Indirect bilirubin reached 2.8 mg/dL on day 3. Total bilirubin was 7.2 mg/dL. Further results are summarised in Table 2 below. The haemolytic nature of the patient’s blood may, however, have influenced some of the parameters. 

Antibody differentiation was negative. No reaction took place when eluting the erythrocytic antibodies. Further haematological results are summarised in Table 3 below.

### 3.2. Post-Mortem Immunohaematological Tests

#### 3.2.1. Case 1

Antibody screening of post-mortem blood tested positive in an enzyme pre-treatment and indirect Coombs test (ICT) with Coombs serum. Our antibody differentiation test was in accordance with results obtained during hospitalisation and confirmed the presence of an irregular erythrocytic antibody with anti-e specificity. Auto-control and DCT demonstrated strong erythrocytic adsorption (4+). Auto-anti-e was differentiated in an ICT-technique after acid elution from the erythrocyte surface. Lefèvre’s mushroom haemagglutination test with extracts from *Paxillus involutus* [11] was positive for agglutination. Control tests of patient blood with erythrocytes from blood lacking the Rhesus antigen-e and with extracts from *Rubroboletus satanas* (Satan’s bolete) were negative for agglutination. Lefèvre’s test was, however, also negative with the sample obtained after antibody elution. 

#### 3.2.2. Case 2

DCT demonstrated strong adsorption of antibodies to erythrocyte surfaces (4+). No irregular erythrocyte antibody was detected during antibody differentiation. Lefèvre’s mushroom haemagglutination test with extracts from *Paxillus involutus* [11] was, however, negative for agglutination. 

### 3.3. Mycological Analyses

All mycological analyses were performed by a mycologist.

#### 3.3.1. Analysis of Stomach Contents of Case 1

**Figure 3:**
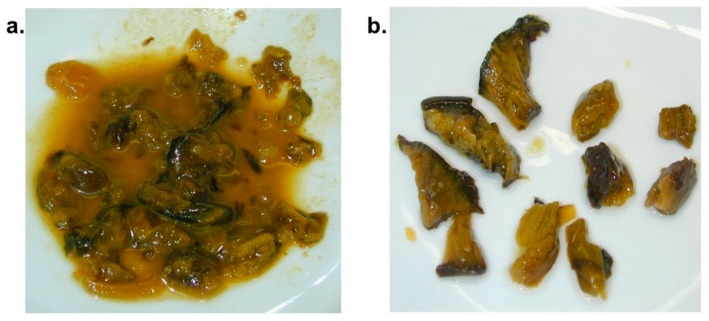
Mushroom residues from stomach contents of case 1 (**a**) before and (**b**) after preparation for analysis.

Mushroom residues were isolated from stomach contents (see Figure 3) and positively identified as *Paxillus involutus*, based on macroscopic and microscopic features.

**Figure 4:**
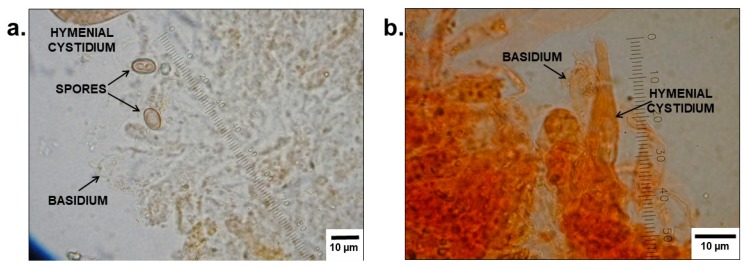
Microscopic images of the lamellar surfaces of mushroom residues in Figure 3, prepared in (**a**) water and (**b**) Congo red, respectively. The spores, basidia, and cystidia of *Paxillus involutus* can be seen.

Several samples were prepared from fluid stomach contents (see Figure 3) and examined by different staining techniques (see Figure 4). No indication for the consumption of other mushroom species could be found.

#### 3.3.2. Analysis of Samples from the Mushroom Meal Consumed by Patient 2

**Figure 5:**
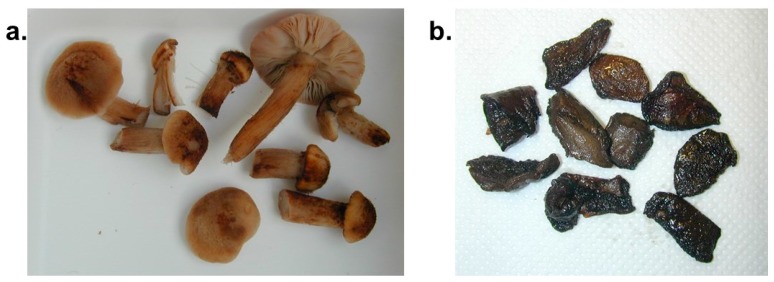
Purified samples of the patient’s last mushroom meal, containing (**a**) Armillaria mushrooms (honey fungi) and (**b**) *Paxillus involutus*.

**Figure 6:**
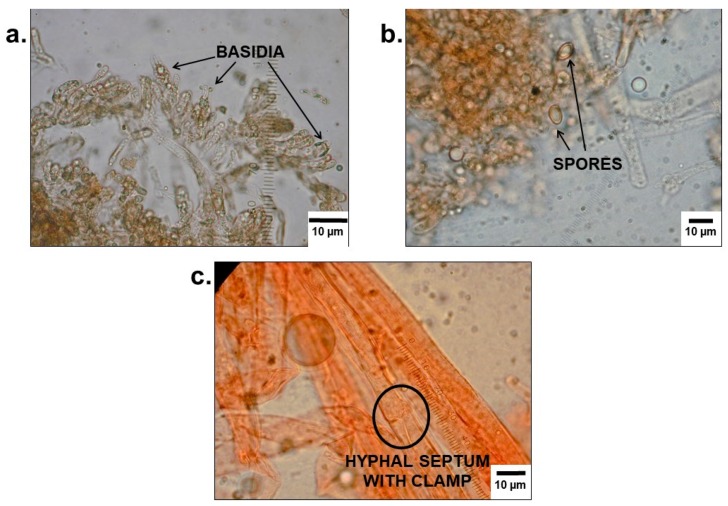
Microscopic images of the samples of *Paxillus involutus*, showing the (**a**) basidia and (**b**) spores in water, as well as a (**c**) hyphal septum with clamp connection in Congo red.

Two mushrooms were identified during macroscopic (Figure 5) and microscopic (Figure 6) analysis of samples from the mushroom meal. Additional to the popular honey fungus, several mushroom cap pieces of *Paxillus involutus* could be found. 

### 3.4. Post-Mortem Examination

#### 3.4.1. Autopsy of Case 1

**Figure 7:**
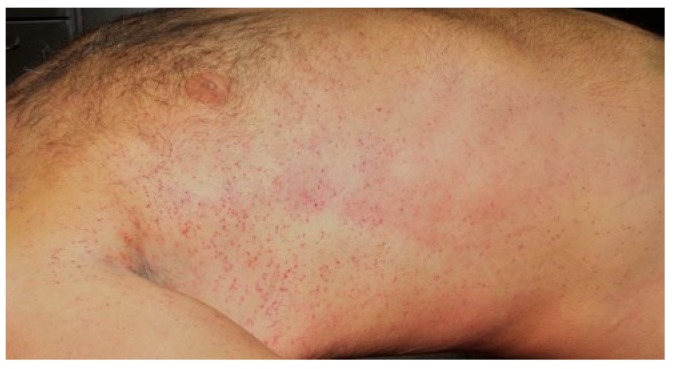
Petechiae located around the torso of patient 1 during post-mortem examination.

Several autoptic findings pointed towards disseminated intravascular coagulation as cause of death. These included petechiae on the skin (see Figure 7), mucous membranes and visceral pleura, countless punctate haemorrhages in the nervous tissue, bleeding sites in the pericardium, endocardium and cardiac muscle, as well as pronounced haemorrhages in the patient’s muscle tissues and areas surrounding injection sites. Furthermore, a splenic rupture could be located, which may, however, also have resulted during resuscitation of the patient during hospital treatment.

#### 3.4.2. Necropsy of Case 2

Haemorrhages in the sclera and possible puncture sites on the left antecubital fossa and left foot were identified during the necropsy. No external injuries relevant to the death of the patient were established. 

The public prosecution authorities did not order an autopsy for case 2.

### 3.5. Toxicological Analyses

Substances detected in bodily fluids of case 1 are presented in Table 4. No alcohol nor anticoagulants (e.g., vitamin K antagonists) were detected during specialised tests.

Substances detected in serum of case 2 are presented in Table 5. Specialised tests for anticoagulants, such as vitamin K antagonists, were negative.

## 4. Discussion

**Figure 8:**
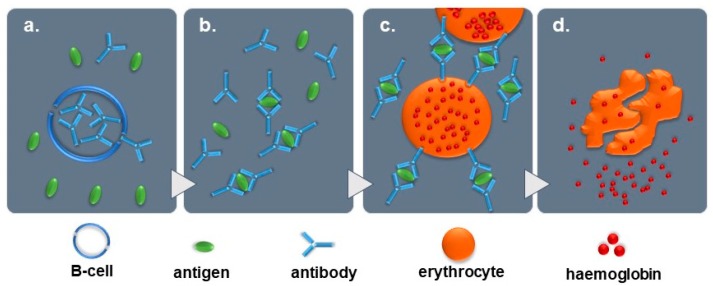
Simplified presentation of the proposed pathophysiological mechanism involving the immunohaemolytic reaction during the Paxillus syndrome. (**a**) Release of antibodies from B-cells, (**b**) antigen-antibody reaction, (**c**) attachment of immune complex to erythrocyte membrane, (**d**) disruption of erythrocytes [8,9,13].

The Paxillus syndrome is a very rare, nevertheless life-threatening medical condition, which is triggered by the consumption of *Paxillus involutus* mushrooms. Partially because no toxin could thus far be identified that causes the AIHA, its diagnosis remains challenging. Furthermore, no certain underlying pathophysiological mechanism has been identified. It has, however, been suggested that an antigen in the mushroom may lead to the formation of antibodies in pre-sensitised consumers. Later contact with the mushroom causes depletion of the antibodies from B-cells, resulting in an antigen-antibody reaction. This immune complex attaches to erythrocyte surfaces, causing the erythrocytes to disrupt and lead to the life-threatening haemolytic reaction (see Figure 8) [8,9,13]. 

When diagnosing the Paxillus syndrome, other causes for the formation of anti-erythrocytic antibodies, such as drug induced haemolytic anaemia (DIHA) [14] or chronic hepatitis C [15] should be considered. In our two cases, none of the drugs detected during routine post-mortem toxicological analysis is associated with DIHA. Furthermore, targeted toxicological analyses for anticoagulants were negative. However, in retrospect, respective findings in the two patients may have made the diagnosis of the Paxillus syndrome as cause of the haemolysis doubtful. Patient 1 possessed an auto-anti-e antibody. Patient 2 suffered from a chronic hepatitis C infection. Furthermore, Lefèvre’s tests for both cases were negative for agglutination (after acid elution of auto-anti-e in case 1), also suggesting another cause for the haemolysis. However, earlier reports show that, depending on the area of collection, not all extracts of *Paxillus involutus* lead to agglutination [7,9,13]. We collected our mushrooms for both tests from the same region. All these factors underline the difficulty in diagnosing the Paxillus syndrome, indicating the high value of a thorough patient anamnesis. The following factors are crucial to consider: The onset of symptoms typically occurs rapidly, approximately two hours after mushroom consumption. Literature shows that all victims of the Paxillus syndrome had repeatedly ingested *Paxillus involutus* in the past. The condition does therefore not occur in first-time consumers, suggesting a foregoing pre-sensitisation towards an antigen in the mushroom. Furthermore, although several persons may have consumed of the same meal, only one person is affected. This is in contrast to the condition which mainly involves gastric side-effects, caused by the same mushrooms [6,7,8,9,13]. Manifestations of acute haemolysis include DIC, severe back and abdominal pain, gastrointestinal symptoms, lactic acidosis and progressive multiorgan failure, predominantly affecting the liver and kidneys. Blood tests typically indicate massive decreases in prothrombin time, haemoglobin and haptoglobin. Liver parameters increase dramatically. The sudden hyperbilirubinaemia is helpful in distinguishing the Paxillus syndrome from amatoxin poisoning. Unconjugated bilirubin rises within a few hours after ingesting *Paxillus involutus*, whereas conjugated bilirubin is elevated only a few days after amatoxin consumption. Mycological identification of the consumed mushroom is another important aid in the diagnosis [2].

In summary, a quick differential diagnosis can be made by a long history of consuming the mushroom by the patient, the rapid onset of symptoms [6], the sudden rise in bilirubin and the absence of symptoms in co-consumers. Pre-existing liver and kidney damage may exacerbate the condition. 

## 5. Conclusions

The auto-anti-e antibody and chronic hepatitis C infection in our two cases may have complicated the diagnosis of the Paxillus syndrome, because these underlying factors can be a direct trigger for haemolysis, even in the absence of the Paxillus mushroom. However, our patients—and perhaps also others—may rather have been more susceptible for this condition due to these underlying factors. It should nevertheless be noted that, from the fourteen published reports, we were able to identify only one other case accompanied by chronic hepatitis C [6] and one with an untypical Rhesus-formula [13]. This warrants more detailed investigation into the mechanism underlying the Paxillus syndrome. 

Nevertheless, it may not be underestimated that the consumption of *Paxillus involutus* poses a high health risk and may even end fatally. The rare prevalence of the Paxillus syndrome makes mushroom hunters unaware of the toxic potential of poison pax, with the mushroom still being consumed regularly. Furthermore, rare diseases such as this one, pose a challenge to health care providers in making the correct diagnosis. This publication provides helpful guidelines for the diagnosis and differential diagnosis of the Paxillus syndrome. Adequate anti-shock therapy and plasmapheresis may be essential for a positive outcome [9].

## Figures and Tables

**Table 1:** Timeline of sequence of events between consumption and death of the two respective patients.

**Patient 1**						
**DAY 1**				**DAY 3**	**DAY 4**	**DAY 5**
**12:00**	**15:00**	**evening**				**18:00**
Mushroom consumption	Severe backache			Respiratory & circulatory complications	Septic shock	Patient declared brain dead
	Hospital admission	Transfer to maximum-care hospital			Sudden hypertension	Exitus letalis
	Increasing abdominal pain	Improvement of liver function			Massive cerebral swelling	
	Acute liver failure					
**Patient 2**						
**DAY 1**				**DAY 2**	**DAY 3**	**DAY 5**
**19:00**	**21:00**	**22:05**	**22:40**	**06:00**		**02:30**
Mushroom consumption	Abdominal pain, nausea & vomiting	Notification of emergency medical services	Hospital admission & treatment Patient stabilised	Transfer to maximum-care hospital Plasmapheresis, kidney replacement & haemo-adsorption filters therapy - Patient stabilised	Patient’s condition worsens Acute on chronic liver failure	Exitus letalis

**Table 2:** Aspartate aminotransferase (AST, GOT), alanine aminotransferase (ALT, GPT) and lactate dehydrogenase (LDH) in serum of patient 2, measured on day 4 of hospitalisation.

Parameter	Levels Measured	Reference Range
AST, GOT	9500 U/L	8–43 U/L
ALT, GPT	2800 U/L	7–45 U/L
LDH	7600 U/L	122–222 U/L

**Table 3:** Results obtained from haematological tests in whole blood of patient 2 during hospitalisation.

Blood Group	B
Rhesus Formula	CcD.ee
**Direct Coombs Test (DCT)**	
Polyspecific Coombs serum	+++
Monospecific Coombs serum with:	
Anti-IgG	+
Anti-C3d	+++

**Table 4:** Substances detected in femoral blood, urine and stomach contents of case 1.

Femoral Blood		Urine	Stomach Contents
substance	concentration	substance	substance
Midazolam	2400 µg/L	Midazolam	Midazolam
Hydroxymidazolam	100 µg/L	Hydroxymidazolam	Hydroxymidazolam
Sufentanil	2.0 µg/L	Sufentanil	Sufentanil
4-Methylaminoantipyrine	not quantified	4-Methylaminoantipyrine	4-Methylaminoantipyrine
Piritramide	1.7 µg/L	Piritramide	not detected
Metoclopramide	64 µg/L	Metoclopramide	Metoclopramide
Lidocaine	not quantified	Lidocaine	Lidocaine
Propofol	not analysed	Propofol	not analysed
not analysed	not analysed	Caffeine	not analysed

Substances in urine and stomach contents were not quantified.

**Table 5:** Substances detected in serum of case 2, obtained on day 2 of hospitalisation.

Patient Serum	
Substance	Concentration
Midazolam	110 µg/L
Hydroxymidazolam	22 µg/L
Lidocaine	44 µg/L
Remifentanil acid	not quantified
Ibuprofen	not quantified
Cotinine	not quantified
Caffeine	not quantified

Remifentanil acid (metabolite of remifentanil), ibuprofen, cotinine (metabolite of nicotine) and caffeine were not quantified.
